# Supplementary material for: From sequence to enzyme mechanism using multi-label machine learning
Source: BMC Bioinformatics. 2014 May 19;15:150. doi: 10.1186/1471-2105-15-150 (PMC4229970; doi:10.1186/1471-2105-15-150)
Supplement: Additional file 2 — Java code of ml2db. Additional file ml2db_code.tar.gz contains the Java source code to run the multi-label machine learning experiments and save the results to database. The code’s Javadoc is included. [file 1471-2105-15-150-S2.zip › additional file 2/ml2db/utils/doc/edu/cornell/lassp/houle/RngPack/Ranlux.html]

Ranlux


JavaScript is disabled on your browser.


- Overview
- Package
- Class
- Use
- Tree
- Deprecated
- Index
- Help

- Prev Class
- Next Class

- Frames
- No Frames

- All Classes

- Summary:
- Nested |
- Field |
- Constr |
- Method

- Detail:
- Field |
- Constr |
- Method


edu.cornell.lassp.houle.RngPack

## Class Ranlux

- java.lang.Object
- - cern.colt.PersistentObject
  - - edu.cornell.lassp.houle.RngPack.RandomElement
    - - edu.cornell.lassp.houle.RngPack.RandomSeedable
      - - edu.cornell.lassp.houle.RngPack.Ranlux

- All Implemented Interfaces:
  :   java.io.Serializable, java.lang.Cloneable

  ---

    

  ```
  public class Ranlux
  extends RandomSeedable
  ```

  RANLUX is an advanced pseudo-random number generator based on the
  RCARRY algorithm proposed in 1991 by Marsaglia and Zaman.
  RCARRY used a subtract-and-borrow algorithm with a period on the
  order of 10^171^ but still had detectable correlations between
  numbers. Martin Luescher proposed the RANLUX algorithm in 1993;
  RANLUX generates pseudo-random numbers using RCARRY but
  throws away numbers to destroy correlations. Thus RANLUX trades execution
  speed for quality; by choosing a larger luxury setting one gets better random
  numbers slower. By the tests availible at the time it was proposed,
  RANLUX at the default luxury setting appears to be a significant
  advance quality over previous generators.
    
    


  |  |  |  |
  | --- | --- | --- |
  | LUXURY LEVELS | | |
  | level | p |  |
  | 0 | 24 | equivalent to the original RCARRY of Marsaglia and Zaman, very long period, but fails many tests. |
  | 1 | 48 | considerable improvement in quality over level 0, now passes the gap test, but still fails spectral test. |
  | 2 | 97 | passes all known tests, but theoretically still defective. |
  | 3 | 223 | DEFAULT VALUE. Any theoretically possible correlations have very small chance of being observed. |
  | 4 389 | highest possible luxury, all 24 bits chaotic. | |  |  |

     
   **VALIDATION** 
  The Java version of RANLUX has been verified against published
  values of numbers 1-5 and 101-105 produced by the reference implementation of
  RANLUX for the following initial conditions:
  - Default initialization: `Ranlux()`- Initialization with: `Ranlux(0,0)`- Initialization with: `Ranlux(389,1)`- Initialization with: `Ranlux(75,0)`
  References:
  - M. Luscher,  Computer Physics Communications **79** (1994)
    100- F. James, Computer Physics Communications **79** (1994) 111- About RANLUX random number generator: Excerpts from discussion in
        the Usenet news groups- Miller's FORTRAN 90 implementation of RANLUX with test code

  Source code  is available.

  Version:
  :   1.0

  Author:
  :   Paul Houle  (E-mail: houle@msc.cornell.edu)

  See Also:
  :   Serialized Form

- - ### Field Summary

    Fields

    | Modifier and Type | Field and Description |
    | --- | --- |
    | `static int` | `lxdflt` Default luxury level: `lxdflt=3` |
    | `static int` | `maxlev` Maximum luxury level: `maxlev=4` |
  - ### Constructor Summary

    Constructors

    Constructor and Description

    `Ranlux()`

    Default initialization of RANLUX.

    `Ranlux(java.util.Date d)`

    Initialize RANLUX with default luxury
    level and a Date object.

    `Ranlux(int ins)`

    Initialize RANLUX with default luxury
    level and a specified seed.

    `Ranlux(int lux,
    java.util.Date d)`

    Initialize RANLUX with specified luxury
    level and a Date object.

    `Ranlux(int lux,
    int ins)`

    Initialize RANLUX with specified luxury
    level and seed.

    `Ranlux(int lux,
    long ins)`

    Initialize RANLUX with specified luxury
    level and seed.

    `Ranlux(long ins)`

    Initialize RANLUX with default luxury
    level and a specified seed.
  - ### Method Summary

    Methods

    | Modifier and Type | Method and Description |
    | --- | --- |
    | `java.lang.Object` | `clone()` Returns a copy of the receiver; the copy will produce identical sequences. |
    | `double` | `raw()` Returns a uniformly distributed pseudo-random double in the range (0,1). |
    | `void` | `setDiag(boolean b)` Turns diagnostic messages on and off. |

    - ### Methods inherited from class edu.cornell.lassp.houle.RngPack.RandomSeedable

      `ClockSeed, ClockSeed`
    - ### Methods inherited from class edu.cornell.lassp.houle.RngPack.RandomElement

      `choose, choose, gaussian, gaussian, powlaw, raw, raw, uniform`
    - ### Methods inherited from class java.lang.Object

      `equals, getClass, hashCode, notify, notifyAll, toString, wait, wait, wait`

- - ### Field Detail


    - #### lxdflt

      ```
      public static final int lxdflt
      ```

      Default luxury level: `lxdflt=3`

      See Also:
      :   Constant Field Values


    - #### maxlev

      ```
      public static final int maxlev
      ```

      Maximum luxury level: `maxlev=4`

      See Also:
      :   Constant Field Values
  - ### Constructor Detail


    - #### Ranlux

      ```
      public Ranlux()
      ```

      Default initialization of RANLUX. Uses default seed
      `jsdflt=314159265` and luxury level 3.


    - #### Ranlux

      ```
      public Ranlux(java.util.Date d)
      ```

      Initialize RANLUX with default luxury
      level and a Date object. Can be used to conveniently initialize
      RANLUX from the clock,

      ```
       RandomElement e = Ranlux(new Date());
      ```

      Parameters:
      :   `d` - date used to generate seed


    - #### Ranlux

      ```
      public Ranlux(int ins)
      ```

      Initialize RANLUX with default luxury
      level and a specified seed.

      Parameters:
      :   `ins` - seed, a positive integer


    - #### Ranlux

      ```
      public Ranlux(int lux,
            java.util.Date d)
      ```

      Initialize RANLUX with specified luxury
      level and a Date object. Can be used to conveniently initialize
      RANLUX from the clock,

      ```
       RandomElement e = Ranlux(4, new Date());
      ```

      Parameters:
      :   `lux` - luxury level from 0-4.
      :   `d` - date used to generate seed


    - #### Ranlux

      ```
      public Ranlux(int lux,
            int ins)
      ```

      Initialize RANLUX with specified luxury
      level and seed.

      Parameters:
      :   `lux` - luxury level from 0-4.
      :   `ins` - seed, a positive integer.


    - #### Ranlux

      ```
      public Ranlux(int lux,
            long ins)
      ```

      Initialize RANLUX with specified luxury
      level and seed.

      Parameters:
      :   `lux` - luxury level from 0-4.
      :   `ins` - seed, a positive long.


    - #### Ranlux

      ```
      public Ranlux(long ins)
      ```

      Initialize RANLUX with default luxury
      level and a specified seed.

      Parameters:
      :   `ins` - seed, a positive integer
  - ### Method Detail


    - #### clone

      ```
      public java.lang.Object clone()
      ```

      Returns a copy of the receiver; the copy will produce identical
      sequences. After this call has returned, the copy and the receiver have
      equal but separate state.

      **Overrides:**
      :   `clone` in class `PersistentObject`

      Returns:
      :   a copy of the receiver.


    - #### raw

      ```
      public final double raw()
      ```

      Returns a uniformly distributed pseudo-random double in the range (0,1).

      **Specified by:**
      :   `raw` in class `RandomElement`

      Returns:
      :   a uniformly distributed pseudo-random double in the range (0,1).

      See Also:
      :   `RandomJava`


    - #### setDiag

      ```
      public void setDiag(boolean b)
      ```

      Turns diagnostic messages on and off. If setDiag(true) is
      called, RANLUX will print diagnostic information to
      System.err

      Parameters:
      :   `b` - diagnostic message status


- Overview
- Package
- Class
- Use
- Tree
- Deprecated
- Index
- Help

- Prev Class
- Next Class

- Frames
- No Frames

- All Classes

- Summary:
- Nested |
- Field |
- Constr |
- Method

- Detail:
- Field |
- Constr |
- Method
